# Not Merely Experiential: Unconscious Thought Can Be Rational

**DOI:** 10.3389/fpsyg.2017.01096

**Published:** 2017-07-06

**Authors:** Katie E. Garrison, Ian M. Handley

**Affiliations:** ^1^Department of Psychology, Texas A&M University, College StationTX, United States; ^2^Department of Psychology, Montana State University, BozemanMT, United States

**Keywords:** unconscious thought, rational and experiential systems, consciousness, problem solving, judgment and decision making

## Abstract

Individuals often form more reasonable judgments from complex information after a period of distraction vs. deliberation. This phenomenon has been attributed to sophisticated unconscious thought during the distraction period that integrates and organizes the information (Unconscious Thought Theory; Dijksterhuis and Nordgren, 2006). Yet, other research suggests that experiential processes are strengthened during the distraction (relative to deliberation) period, accounting for the judgment and decision benefit. We tested between these possibilities, hypothesizing that unconscious thought is distinct from experiential processes, and independently contributes to judgments and decisions during a distraction period. Using an established paradigm, Experiment 1 (*N* = 319) randomly induced participants into an experiential or rational mindset, after which participants received complex information describing three roommates to then consider consciously (i.e., deliberation) or unconsciously (i.e., distraction). Results revealed superior roommate judgments (but not choices) following distraction vs. deliberation, consistent with Unconscious Thought Theory. Mindset did not have an influence on roommate judgments. However, planned tests revealed a significant advantage of distraction only within the rational-mindset condition, which is contrary to the idea that experiential processing alone facilitates complex decision-making during periods of distraction. In a second experiment (*N* = 136), we tested whether effects of unconscious thought manifest for a complex analytical reasoning task for which experiential processing would offer no advantage. As predicted, participants in an unconscious thought condition outperformed participants in a control condition, suggesting that unconscious thought can be analytical. In sum, the current results support the existence of unconscious thinking processes that are distinct from experiential processes, and can be rational. Thus, the experiential vs. rational nature of a process might not cleanly delineate conscious and unconscious thought.

## Introduction

People can rely on a variety of processes to guide their decisions and judgments regarding complex information. For example, one might think carefully and rationally, or alternatively “feel out” information to arrive at a conclusion. Indeed, many dual-process models in psychology propose that judgments and decisions can predominantly result from either analytical or experiential/emotional processing systems (e.g., Epstein, 1994; Sloman, 1996; Kahneman, 2003; see Evans, 2008 for review). Of current focus, Cognitive Experiential Self Theory (CEST; Epstein, 1994, 1998) describes two parallel information-processing systems – an experiential system and a rational system – that contribute to judgments and decisions. These two systems are global aspects of personality, and within each system are psychological processes. Within the experiential system are automatic, fast, intuitive, holistic, pre-conscious and emotional processes. Within the rational system are effortful, deliberate, slow, rational and conscious processes. Furthermore, a relative reliance on one system over the other is a trait-like individual difference (Epstein et al., 1992) and can be manipulated in the laboratory (e.g., Krauss et al., 2004). For instance, an individual with a relatively dominant experiential system may make judgments and decisions based on a “vibe” or hunch, paying close attention to his or her emotions, whereas an individual with a more dominant rational system may follow rules and careful analysis to reach a decision.

The general assumption underlying CEST, and many other dual-process models, is that the rational system operates on a conscious level, whereas the experiential system operates more unconsciously (Evans, 2008). Researchers commonly use factors like the speed, or the evident rational vs. emotional basis of a decision as clues that conscious or unconscious processes produced the response. In this paper, we challenge the implicit or explicit assumptions undergirding this practice, and hope to refine the attributes distinguishing conscious and unconscious processes.

To this end, we offer that *unconscious thought*, as proposed by Unconscious Thought Theory (UTT; Dijksterhuis and Nordgren, 2006; Dijksterhuis and Strick, 2016) resembles both experiential and rational modes of processing, and therefore does not fit neatly within a two-system framework. As described in more detail below, unconscious thought operates outside of conscious awareness, but does so over a period of time (i.e., not quickly and automatically), and in a goal-directed manner. These features of unconscious thought resemble the rational mode, and we therefore propose that unconscious thought may be independent of the two modes of processing. The current research presents two experiments testing the hypothesis that unconscious thought is distinct from purely experiential processes and can be rational.

## The MIS-FIT of Unconscious Thought

Much research and theorizing support an unconscious mode of processing that does not fit neatly within a two-system framework. In particular, UTT asserts that unconscious processes can integrate, organize, and evaluate complex information (i.e., much and varied information) over several minutes to facilitate sound judgments and decisions (Dijksterhuis and Nordgren, 2006; Dijksterhuis and Strick, 2016). Dijksterhuis and Nordgren (2006, p. 96) call this process *unconscious thought*, defining it as “object-relevant or task-relevant cognitive or affective thought processes that occur while conscious attention is directed elsewhere.” Unconscious thought is presumed to be goal-directed, occur over a period of time, and facilitate decision-making by actively organizing and processing information (Bos et al., 2008). It is akin to incubation (e.g., Smith and Blankenship, 1991) or “mulling over” information below the threshold of conscious awareness.

In a typical UTT experiment (e.g., Dijksterhuis, 2004), participants receive complex information about various options (e.g., apartments), are asked to form impressions from the information and ultimately evaluate the options or choose the best one. Commonly, there are 4 options associated with 12 attributes each, some positive and some negative, totaling to 48 pieces of information. Participants view each piece of information randomly and one at a time (e.g., “Apartment A is in a nice area,” “Apartment B has an unfriendly landlord,” etc.). The information is rigged such that one option has more positive attributes and fewer negative attributes than the others (e.g., eight positive and four negative), one has more negative attributes and fewer positive attributes than the others (e.g., eight negative and four positive), and two have an equal number of positive and negative attributes (six positive and six negative).^1^ After information acquisition, participants are randomly assigned to thought conditions. In the *unconscious-thought condition*, participants engage in a distraction task for 3 or 4 min (e.g., an anagram or working memory task), during which they are unable to consciously think about the choice options. In the *conscious-thought condition*, participants are instructed to consciously deliberate on the information for the same duration. Further, many such experiments also include a control condition, like an *immediate-choice condition* in which participants make their judgments and choices immediately following information acquisition (i.e., leaving negligible time for conscious or unconscious thought; Dijksterhuis, 2004), or a *mere-distraction condition* in which participants are disabused of the goal to process the information just after it is presented, then engage in a distraction task (Bos et al., 2008). Finally, participants rate the options, or choose an option. Results from such designs commonly show that participants in the unconscious thought condition (relative to other thought conditions) form more favorable judgments toward the best option and less favorable judgments toward the worst option, or are more likely to choose the best option. One interpretation of this effect is that conscious capacity is limited and therefore cannot effectively process all of the relevant information (e.g., 48 attributes), whereas unconscious capacity is much larger and can handle the quantity of information to form reasonable judgments (Dijksterhuis and Nordgren, 2006).

Past research has revealed that participants in unconscious thought conditions, relative to conscious thought or control conditions, integrate and evaluate considerable information (e.g., Dijksterhuis, 2004; Dijksterhuis et al., 2006; Bos et al., 2008; Lerouge, 2009; Ham and van den Bos, 2011; Manigault et al., 2015), weight information based on subjective importance (Bos et al., 2011), and recall information in a more organized fashion and clustered around similar traits (Bos et al., 2008). According to UTT, these patterns reflect a sophisticated unconscious thought process capable of integrating and organizing complex information. Of note, this pattern of results actually represents two different effects: an unconscious thought advantage (UTA; Nieuwenstein et al., 2015) relative to conscious-thought conditions, and a true unconscious thought effect (UTE) relative to control conditions such as mere distraction and immediate choice. Regardless, many interpret these patterns as evidence that unconscious thought can evaluate and process complex information in a goal-directed, slow, and seemingly analytical manner; qualities typically associated with rational (or conscious) processes in the CEST and related dual-process models.

Although many experiments and a meta-analysis reported by Strick et al. (2011) support the existence of the UTA and UTE, these effects remain somewhat controversial. Indeed, several papers also report failures to replicate, and a very recent meta-analysis and well-powered replication found no evidence for the UTA in choices using the type of information presentation described above (Nieuwenstein et al., 2015). For the present purposes, we offer that inconsistent results likely suggest unrevealed psychological moderators to the effects, and recommend well-powered experiments continue to test UTT.

## Is the Unconscious Thought Effect Merely the Result of Intuitive/Experiential Processes?

Replications and meta-analyses aside, some researchers have proposed alternative explanations for the effects taken to support UTT (e.g., Lassiter et al., 2009; Usher et al., 2011; Nieuwenstein et al., 2015). Focal to the current paper, Usher et al. (2011) suggest that the decision benefit observed following a period of distraction vs. deliberation is not due to unconscious thinking *per se*, but to a reliance on the experiential processing system. They reasoned that “decisions performed in the distraction condition rely to a higher degree on intuitive strategies than decisions performed after deliberation” (p. 2). That is, the period of distraction relative to deliberation was thought to dampen down influences of the rational system and heighten the activation of the experiential system. This relative reliance on the experiential system should then impact how individuals process subsequent information (i.e., in a more holistic, emotional, intuitive manner). So, with a more active experiential system, individuals may “go with their gut” or use emotion or intuition to guide their choices.

In several experiments, Usher et al. (2011) use manipulations based on CEST (Epstein, 1994) to heighten the experiential system or the rational system. In one experiment, participants were induced into either an experiential mindset by focusing on and drawing their current emotional state for 3 min, or a rational mindset by solving math problems for the same duration. Next, participants made a complex decision about different car options. In line with their predictions, results revealed that participants primed with the experiential mindset chose the best car option more frequently and reported greater attitude differentiation between the best and worst cars (i.e., better evaluations overall) compared to participants primed with a rational mindset. These results support the notion that emotion-based decisions can be advantageous when the information is sufficiently complex.

In a follow-up experiment, Usher et al. (2011) added in a manipulation of thought (distraction vs. deliberation) used in typical UTT experiments to elicit unconscious vs. conscious thought, respectively. This was done to “maximize the difference between intuitive vs. analytic modes of thought” (p. 8), under the assumption that distraction would strengthen the experimental system and deliberation would strengthen the rational system. In Experiment 4, participants were induced into either a rational or experiential mindset as before, prior to receiving complex information describing three different roommate options (where one was objectively the best, one was objectively the worst, and one was neutral based on attribute qualities). The manipulation of thought (distraction vs. deliberation) was paired with the manipulation of mindset such that participants who were primed into a rational mindset additionally deliberated on the options, whereas participants who were primed into an experiential mindset were additionally distracted with anagrams. Results from this study revealed that participants in the distraction/experiential mindset condition rated the best roommate more favorably relative to participants in the deliberation/rational mindset condition, and had greater attitude differentiation scores between the best and worst roommates (i.e., overall evaluation). The authors suggested that this decision benefit resulted from processes in the experiential mode (they used the terms *intuitive* and *affective*) that were strengthened during the distraction period, rather than an active unconscious thought process independent of the two mindsets.

However, this experimental design did not allow for a clear test between the effects of unconscious thought and an experiential mindset on decision outcomes. The modes of processing (rational vs. experiential) and thought condition (conscious vs. unconscious) were confounded. In Experiment 4, only participants who were distracted from thinking about the information (i.e., unconscious thought condition) were placed in the experiential mindset, and only participants who consciously thought about the options were placed in the rational mindset (Usher et al., 2011). Logically then, it is impossible to know whether the observed difference was due to mindset, thought modality, or an additive effect due to redundant manipulations. To truly test whether the experiential processing mode is responsible for the decision benefit following distraction, it is necessary to conduct a fully crossed design in which mindset and thought modality are manipulated independently. We investigated exactly that design in our Experiment 1. As it stands, it is possible that individuals will form better decisions when they are distracted, rather than when they think consciously, even if (or especially if) they are in an *analytical* mindset.

## Research Question and Overview of Experiments

Our research question is whether the experiential processing mode is solely responsible for the UTA and UTE observed following a period of distraction, or whether unconscious thought is orthogonal to experiential and rational modes. Ultimately, the answer to this question can help us understand whether the analytic or emotional nature of a process gives us clear insight into whether that process was conscious or unconscious.

We conducted two experiments to investigate our research question. In the first experiment, we largely replicated Usher et al.’s (2011) Experiment 4, except we randomly assigned participants to experience a rational or experiential mindset prior to receiving complex information describing three different roommates, *and* randomly assigned participants to deliberate on the roommate options or complete anagrams as a distraction during information acquisition.^2^ If the UTA is merely the result of a dominant experiential system, then we should observe an advantage in roommate judgments and choices only among participants with an experiential mindset (as observed in Usher et al., 2011). Alternatively, if the UTA is driven by unconscious thought and independent from the experiential mode, we should observe the advantage in roommate judgments and choices in the unconscious-thought condition regardless of mindset.

In the second experiment we presented participants with a complex logical reasoning problem, then randomly assigned participants to adopt the goal (unconscious thought condition), or not (control condition) to solve that problem prior to a distraction task. Participants then reported solutions to this problem after the distraction task. The experiential system should offer no assistance in solving a complex logical reasoning problem (which would require the rational system). Therefore, if we observe an effect of unconscious thought (i.e., UTE) it would suggest that unconscious thought can process analytical information, and is likely independent from the experiential system.

## Experiment 1

We replicated the methods from Usher et al. (2011) and participants were induced into either a rational or experiential mindset before receiving information describing three different roommates. They were asked to form impressions of the roommates and ultimately make a decision about which one they would most like to live with. During the acquisition of the roommate information, participants either deliberated on reasons for liking or disliking each option (i.e., conscious thought) or they were distracted by solving anagrams (i.e., unconscious thought). We predicted that the experiential vs. rational mindset would lead to better judgments and decisions of the roommate options, consistent with Usher et al. (2011). We also predicted that a period of unconscious vs. conscious thought would lead to better judgments and decisions of the roommate options, consistent with UTT (Dijksterhuis and Nordgren, 2006). Critically, we predicted that unconscious thought would facilitate roommate judgments and decisions *regardless* of the initial mindset. That is, we predicted the emergence of a UTA independent of the mindset. This finding would support the existence of an unconscious thought process that is distinct from the experiential system.

### Method

#### Participants and Design

Three hundred nineteen undergraduate students^3^ from Montana State University participated in the experiment for partial course credit. This experiment was approved by the Institutional Review Board of Montana State University, and participants gave their informed consent before participating. Participants were randomly assigned to the conditions of a 2 (Mindset: rational vs. experiential) × 2 (Thought: conscious vs. unconscious) between-subjects design.

#### Materials

##### Roommate descriptions

The behavioral descriptions for the roommates can be found in Appendix A. They consist of the same 12 binary attributes (i.e., good/bad versions of the same trait) for each of three roommates (e.g., “Roommate A is a bit uptight” vs. “Roommate B is a relaxed and easygoing person”). Because we used the same binary attributes across all three roommates, we were somewhat able to control the relative desirability of each roommate option. The best roommate (i.e., the one presented most positively) had eight positive attributes and four negative ones, the worst (i.e., the one presented most negatively) had eight negative attributes and four positive ones, and the middle option had six positive and six negative attributes. Furthermore, the attributes were pre-tested on levels of importance to exclude extreme attributes that might unfairly bias the options (see Usher et al., 2011). In the current experiment Roommate B was the best option, Roommate C was the worst option, and Roommate A was the middle option (this was randomly determined).

##### Mindset manipulation

We used the same mindset manipulations as Usher et al.’s (2011) Experiment 2. Specifically, participants in the *rational mindset condition* solved math problems on paper for 3 min. The numeric values ranged from 2 to 3 digits (e.g., 24 × 153) and participants likely needed to work out the problems by hand. No calculators were allowed. Participants in the *experiential mindset condition* spent 3 min drawing a picture of their current emotional state. Instructions for this manipulation are as follows: “Feelings and attitudes can be expressed through a number of measures including creative expression. We would like you to draw a picture that describes your gut-level feelings about your emotional state right now” (Usher et al., 2011; p. 5; see Krauss et al., 2004 for the same manipulation). Instructions remained on the screen for the entire 3 min duration, and we provided participants with crayons, pens and paper. These manipulations are based on Epstein’s (1994) CEST and are thought to manipulate a reliance on the emotion-based experiential system and the analytical rational system, respectively.

##### Thought manipulation

**FIGURE 1:**
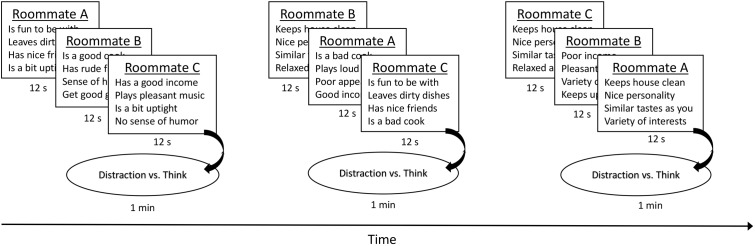
Depiction of roommate information presentation interspersed with thought manipulations. Participants viewed a screen of four roommate descriptions for 12 s before the next screen appeared with four descriptions of the next roommate. After three screens (and three roommates), participants engaged in the thought manipulation [distraction vs. deliberation (think)] for 1 min. Then the cycle repeated. There were three cycles in total.

Most experiments examining UTT randomly assign participants to various thought conditions after they receive all the decision information. However, some experiments, like Experiment 4 of Usher et al. (2011), randomly assigned participants to receive the decision information interspersed with either a distraction task or deliberation periods (this is thought to be more ecologically valid). We elected to replicate Usher et al.’s methodology closely, and thus also interspersed the thought manipulation with the presentation of decision attributes. Specifically, participants received a cycle of 12 roommate descriptions (four descriptions per roommate; a mix of positive and negative), blocked per option, which was followed by either a period of distraction or deliberation (see **Figure 1**). Participants in the *conscious thought condition* deliberated on the options and were asked what they liked or disliked about each of the roommates, one roommate at a time. Participants in the *unconscious thought condition* were distracted and were given three anagrams to solve, one at a time (e.g., DAGERN is an anagram for GARDEN or DANGER).^4^ The cycle of roommate descriptions and thought manipulation occurred three times, which results in approximately 3 min of distraction or deliberation time in total (see **Figure 1**).

##### Task

Participants received computer instructions indicating they would be presented with information describing three potential roommates, and they were to form impressions of the roommates based on the information and ultimately choose the best one to live with. The task consisted of participants reading 12 behavioral descriptions for each of the three roommates (e.g., “Roommate A has a good sense of humor”) totaling to 36 pieces of information.

The presentation of the information was borrowed from the procedures of Usher et al. (2011) Experiment 4. Attributes were blocked per roommate option, such that four behavioral descriptions of the first roommate were presented on screen for 12 s followed by four behavioral descriptions of the second roommate for 12 s, followed by four behavioral descriptions of the third roommate for 12 s (see **Figure 1**). The order in which roommates were presented was randomly determined, but the order of attributes within each roommate was constant and the same for each condition. The order of attribute presentation for each roommate was as follows: Roommate B (best option) displayed 1 positive (+) and 3 negative (1) attributes in the first cycle, 4+ and 0- in the second cycle, and 3+ and 1- in the third cycle (totaling 8+ and 4-). Roommate C (worst option) displayed 2+ and 2- attributes in the first cycle, 0+ and 4- in the second cycle, and 2+ and 2- in the third cycle (totaling 8- and 4+). Roommate A (middle option) displayed 2+ and 2- attributes for each of the three cycles (totaling 6+ and 6-). Please see a complete list of attributes, in the order in which they were presented, in Appendix A.

Following the initial cycle of roommate information presentation (i.e., the first four descriptions of each roommate), participants were randomly assigned to deliberate on the roommate options (conscious thought condition) or to a distraction (unconscious thought condition). After the second round of roommate information presentation, participants again deliberated on the options or were distracted with anagrams (depending on condition). The entire task consisted of three cycles of roommate descriptions (12 attributes for each roommate; 36 attributes total) and three 1-min thought periods after each cycle of either deliberation or distraction.

#### Procedure

Up to six participants entered the laboratory per session and sat at individual computer stations. Participants first engaged in the mindset manipulation, determined via random assignment. Paper, pens, pencils, and crayons were present at each computer for participants to use; instructions for the manipulation were presented on the computer. This manipulation lasted 3 min. Next, participants received computer instructions indicating they would be presented with information describing three potential roommates, and they were to form impressions of the roommates based on the information and ultimately choose the best one to live with. At this point participants engaged in the roommate task (described above). The roommate task lasted roughly 10 min. Following this, participants were asked to rate each roommate on a scale of likeability (1 = *dislike very much*; 10 = *like very much*) and then were asked to choose one of the three roommates with whom they would most like to live. Finally, participants were thanked for their time, debriefed, and dismissed.

### Results

#### Roommate Attitude

The individual roommate ratings were analyzed using a repeated-measures Analysis of Variance (ANOVA), which demonstrated the predicted likeability scores. Overall, participants rated the best option, Roommate B, most likeable (*M* = 6.69, *SD* = 1.85), the worst option, Roommate C, least likable (*M* = 4.45, *SD* = 1.77), and the middle option, Roommate A, in between [*M* = 5.26, *SD* = 1.81; *F*(2,636) = 118.54, *p* < 0.001, $\eta_{p}^{2}$ = 0.272]. All three pairwise comparisons were significant (*p* < 0.001).

The primary dependent measure was an attitude difference score, calculated by subtracting the likeability score of the worst roommate from the likeability score of the best roommate (e.g., attitude BEST – attitude WORST). Larger values of this difference score reflect *greater differentiation* between the best and worst options; that is, more favorable attitudes toward the best roommate option and less favorable attitudes toward the worst roommate option. This difference score in the evaluation measure is typically used in the unconscious thought literature and reflects a global evaluation of the options (e.g., Dijksterhuis, 2004).

The attitude difference score was subjected to a 2 (Mindset: rational vs. experiential) × 2 (Thought: conscious vs. unconscious) between-subjects factorial ANOVA. The analysis yielded no main effect of mindset, *F*(1,315) = 0.213, *p* = 0.644, indicating attitudes toward the best vs. worst roommates were similar in the rational (*M* = 2.31, *SD* = 2.81) and experiential (*M* = 2.17, *SD* = 2.67) mindset conditions. This is inconsistent with the findings of Usher et al. (2011). We did, however, observe a main effect of thought, *F*(1,315) = 6.58, *p* = 0.011, $\eta_{p}^{2}$ = 0.02, such that participants in the unconscious-thought condition reported greater attitude differentiation (*M* = 2.61, *SD* = 2.79) than participants in the conscious-thought condition (*M* = 1.85, *SD* = 2.64). This finding is consistent with UTT, such that a period of distraction that prevents conscious deliberation facilitates the processing of complex information.

**FIGURE 2:**
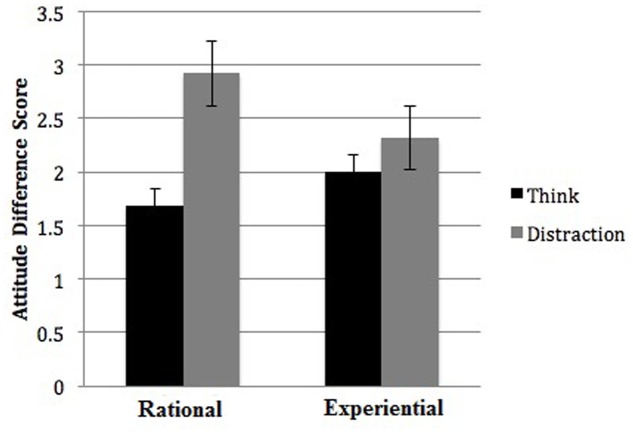
The mean altitude difference scores for roommate options (best – worst) with mindset on the horizontal axis and mode of thought during information acquisition (think vs. distraction) represented by bars. Error bars represent standard errors.

The analysis did not yield a significant interaction between mindset and thought, *F*(1,315) = 2.243, *p* = 0.135, suggesting the UTA was comparable under both mindset conditions. However, we conducted simple-effect tests of thought at each level of mindset to test the competing predictions of Usher et al. (a UTA only within experiential conditions) and our prediction that the UTA is independent of the experiential mode and can occur under rational mindsets as well. Inconsistent with Usher et al.’s predictions and earlier findings, the effect of thought was non-significant in the experiential-mindset condition, *F*(1,315) = 0.59, *p* = 0.443, but was significant in the rational-mindset condition, *F*(1,315) = 7.98, *p* = 0.006, $\eta_{p}^{2}$ = 0.049. Those who solved math problems at the beginning of the experiment (rational mindset) developed superior roommate judgments when they were distracted (*M* = 2.92, *SD* = 2.86) rather than when they deliberated (*M* = 1.68, *SD* = 2.63), Cohen’s *d* = 0.45. The mean attitude difference scores by thought condition and mindset are presented in **Figure 2**.

#### Roommate Choice

Next we assessed the choices participants made about which roommate they wanted to live with. Recall that Roommate B was the best option, Roommate C was the worst option, and Roommate A was the middle option. Overall, 71 participant chose roommate A (22.3%), 212 participants chose roommate B (66.5%), and 36 participants chose roommate C (11.3%). These numbers descriptively match what we predicted.

In order to test whether initial mindset or mode of thought influenced the roommate choices, we collapsed the frequencies of choosing the non-optimal choices (Roommates A and C) into a single *incorrect choice*, and the frequencies of choosing the best choice (Roommate B) into a *correct choice* variable. Then, we analyzed this dichotomous choice outcome (1 = correct, 0 = incorrect) in a logistic regression with the mindset condition, thought condition, and the Mindset × Thought interaction as predictors. The main effect of mindset (rational mindset as reference) was not significant, *B* = 0.28, *SE* = 0.33, *Wald* = 0.72, *p* = 0.396, odds ratio = 1.34, 95% CI [0.69, 2.55], which is reflected in the relative percentages of choosing the correct (vs. incorrect) roommate option for the experiential mindset condition (67.9%) in comparison to the rational mindset condition (64.9%). The main effect of thought (conscious thought condition as reference) was also non-significant, *B* = 0.50, *SE* = 0.34, *Wald* = 2.15, *p* = 0.143, odds ratio = 1.65, 95% CI [0.85, 3.21], yet the pattern of results trended toward a more frequently chosen correct option in the unconscious thought condition (70.1%) vs. the conscious thought condition (62.6%). The interaction between mindset and thought condition was also non-significant, *B* = -0.32, *SE* = 0.48, *Wald* = 0.45, *p* = 0.505, odds ratio = 0.73, 95% CI [0.29, 1.85]. In sum, although participants chose the best roommate option more frequently than the worst, neither initial mindset nor mode of thought significantly influenced this choice.

### Discussion

The results of Experiment 1 support UTT, such that participants who were distracted from thinking about the roommate options formed attitudes that better differentiated between the best and worst options relative to participants who consciously deliberated on them (i.e., the UTA). Interestingly, and inconsistent with the results from Usher et al. (2011), we found no effect of mindset on roommate attitudes. Further, *post hoc* tests on our data revealed no evidence of a significant difference between the experiential/unconscious thought condition and the rational/conscious thought condition, *t*(315) = 1.498, *p* = 0.135; conditions that directly corresponded to Usher et al.’s Experiment 4.

The current study had a large sample size (*N* = 319, averaging 80 participants across four conditions) relative to Usher et al.’s Experiment 4 (*N* = 29, averaging 14–15 participants across two conditions), and thus more power. In their experiment, the difference in attitude scores between the experiential/unconscious thought and the rational/conscious thought conditions yielded an effect size of about 0.819 standard deviation units (e.g., Cohen’s *d*), which is considerably large. Given our sample size, we had 80% power to detect an effect size of *d* = 0.314, which is much smaller. Therefore, we had the statistical power to detect a relatively small difference in attitudes between the experiential/unconscious thought and the rational/conscious thought conditions, yet we found none. Furthermore, by fully crossing the experimental design we were able see a more complete picture of the effects of rational and experiential modes and of unconscious/conscious thought processes on judgments of complex information. The effects of unconscious thought were orthogonal to those of the experiential and rational modes.

Thought and mindset did not interact to influence attitudes, indicating the benefit of distraction was comparable for participants in both the experiential and rational mindset conditions. However, further inspection using simple-effect tests to test competing predictions revealed that the UTA was only significant in the rational-mindset condition (see **Figure 2**). Cumulatively, then, our attitude results support the UTA, and suggest that unconscious thought is not redundant with processes in the experiential mode. Furthermore, they hint that unconscious thought can operate via the rational system. However, we found no evidence that mindset or thought condition influenced roommate choices, meaning participants’ roommate attitudes did not translate into actual decisions.

One goal of the current research was to extend the research of Usher et al. (2011) and test whether unconscious thought is distinct from processes in the experiential mode. Results of Experiment 1 support this notion. Another goal of our research was to determine whether unconscious thought could be rational. There was evidence for this possibility in Experiment 1, as the UTA was largest when paired with a rational mindset. Experiment 2 provided a stricter test of this hypothesis; we gave participants a complex logical-reasoning problem to solve, and an opportunity to think unconsciously about the problem (distraction with a processing goal) or not (mere distraction without a goal). Using a control condition of distraction without a goal allowed us to test for possible effects of unconscious thought over a baseline. A benefit of unconscious thought over mere distraction in the logical reasoning problem would support the existence of a UTE and the hypothesis that unconscious thought can process information analytically. Support for this hypothesis would also strengthen the notion that unconscious thought is independent from the experiential mode (which theoretically should not guide logical reasoning).

## Experiment 2

Similar to traditional unconscious-thought experiments, participants in Experiment 2 received a sequence of complex information to consider (e.g., Dijksterhuis, 2004). However, this information is typically a set of affectively laden (good/bad) attributes describing roommates, cars, vacation destinations, etc. Instead, the current experiment used conditional statements in a logical-reasoning problem as our stimulus material, and these statements did not contain positive or negative connotations. If the emotion-based experiential system facilitates value integration and the weighting of good/bad qualities, it should offer little help in solving a logical-reasoning problem. The current logic problem was a complex deductive reasoning problem consisting of eight sentences and inherent rules to follow. We reasoned that if unconscious thought can be analytical then it should be able to integrate, organize, and evaluate information to deduce logical conclusions.

### Method

#### Participants and Design

One hundred thirty-six undergraduate students from Montana State University participated in the experiment for partial course credit. This experiment was approved by the Institutional Review Board of Montana State University, and participants gave their informed consent before participating. Participants were randomly assigned to one of three conditions: an unconscious thought condition (UT) and a mere distraction condition (MD) for our primary experimental comparison, and additionally a baseline condition in which participants solved the problem out right to determine base rates for solving the problem. The critical comparison to test our hypothesis was between the UT and MD conditions.

#### Procedure

Up to six participants entered the laboratory at a time and sat at individual computer stations. The entire experiment lasted approximately 30 min. Participants were informed that the purpose of the experiment was to investigate problem solving skills and that they would be exposed to a number of different types of problems. Importantly, participants were told that they might be asked to solve some problems or not, intentionally leaving some ambiguity regarding the goal of the experiment.

##### Logical reasoning practice

First, participants were introduced to the *n*-back task of working memory (Jonides et al., 1997). In this task, participants were presented with a random series of single-digit numbers in the center of the computer screen and had to press the space bar whenever a number appeared that had appeared two presentations earlier (e.g., 3, 7, 3). Participants were told that this task was just one of the many problem-solving tasks in the experiment, but it actually served as practice for the distraction task used later in the experiment. Participants engaged in the initial *n*-back for approximately 1 min.

Next participants were instructed to put on headphones and they subsequently learned about logical reasoning. They read instructions and listened to an audio recording over the headphones about the nature of logical reasoning. The recording explained that logical reasoning consists of drawing conclusions from given statements, and presented two simple examples (e.g., “If A is greater than B, and B is greater than C, is A greater than C?”). Following this introduction, participants were given a more complex logical-reasoning problem and were told they may or may not be asked to solve it later, and so must pay close attention.

##### Logical reasoning main task

The main task of the experiment was adapted from the Law School Admissions Test (LSAT) sample questions in the *analytical reasoning* section (Law School Admissions Council, Inc., 2012).^5^ The problem consisted of eight conditional statements that relate to one another. Participants received the problem one sentence at a time, presented visually on the computer screen and audibly via headphones. The presentation of information was very similar to a typical unconscious-thought experiment in which participants receive pieces of information one at a time and must integrate them (e.g., Dijksterhuis, 2004). The problem involved a student needing to perform six different activities on a particular Saturday (e.g., grocery shopping, hedge trimming, jogging, kitchen cleaning, laundry, and motorbike servicing). In the logic problem, the protagonist needed to perform each activity once and in a particular order. The order of the activities was conditional (e.g., laundry must occur before hedge trimming). Therefore, deductive reasoning was required to determine the correct order of activities.

After being presented with the logic problem, participants saw a screen that said, “An example of a question that can be drawn from the preceding statements is ‘*If jogging follows grocery shopping, then which activity must occur first*’? which was followed by the six options. Importantly, participants were not asked to solve the problem, but were ostensibly shown it as an example of a possible problem. At this point, all participants had an identical opportunity to encode the information they needed to solve the problem (i.e., the conditional statements and problem itself) but did not yet have a goal to solve it.

##### Goal manipulation

Participants in the *unconscious-thought condition* (UT, *n* = 68) were told that they would have to solve the logical reasoning problem later in the experiment, and to keep it in the back of their mind. Participants in the *mere-distraction condition* (MD, *n* = 68) were not given a goal to solve the problem. Rather, they were asked a simple question about the information: *Is it true that all activities could occur on a single day?* (Yes or No). Participants in the mere distraction condition answered this question to ensure that no goal to solve the logic problem remained during the upcoming distraction period.

After information acquisition and the goal manipulation, participants in the UT and MD conditions engaged in the *n*-back task again as a distraction task for 3 min. Finally, participants were presented with the original question that appeared at the end of the logic problem earlier in the experiment (*If jogging follows grocery shopping, then which activity must occur first?*), accompanied by each of the answer options. Participants were encouraged to guess if they did not know the answer. Importantly, participants the UT condition expected to receive this problem again at some point in the experiment, but did not know when (hence they had a goal to solve it), but participants in the MD condition did not have this goal. Both groups, however, were distracted from thinking about the problem during the 3 min *n*-back task.

Recall that we included a baseline condition to assess the base rate for solving this problem. Participants in this condition (*n* = 70) received the same information as the UT and MD conditions, but were simply given the question at the end and time to solve it (they were not distracted with the *n*-back). This allowed us to assess the base-rates for solving the logical reasoning problem under normal conditions, and confirm that it was in fact a complex problem.

### Results

For the dependent measure we created a dichotomous variable indicating whether the participants correctly chose the right answer (*correct* = 1) or chose one of the other five options (*incorrect* = 0) as the answer. In this particular example, the correct answer was D, kitchen cleaning. The frequencies with which participants chose each option are presented in **Table 1**. Of note, participants in the base-rate condition chose the correct option 50% of the time, suggesting problem was challenging and difficult to solve under normal test-taking situations.

**Table 1:** Frequencies of choosing each option for the logical reasoning problem in Experiment 2, separated by thought condition: unconscious thought (UT), mere distraction (MD), and baseline (Base).

	*N*	Jogging	Grocery shopping	Hedge trimming	Kitchen cleaning	Laundry	Motorbike servicing
UT	68	6 (8.8%)	20 (29.4%)	2 (2.9%)	17 (25%)	9 (13.2%)	14 (20.6%)
MD	68	10 (14.7%)	25 (36.8%)	1 (1.5%)	8 (11.8%)	7 (10.3%)	17 (25%)
Base	70	2 (2.9%)	20 (28.6%)	3 (4.3%)	35 (50%)	2 (2.9%)	8 (11.4%)


We analyzed the dichotomous dependent measure using a chi-square test of independence between the UT and MD groups. Results indicated that the frequency of choosing the correct vs. incorrect option depended significantly on whether participants were in the UT condition or the MD condition, *X*^2^(1, *N* = 136) = 3.97, *p* = 0.046. Participants in the UT condition correctly solved the logic problem more frequently (17 times or 25% of participants) than participants in the MD condition (8 times or 11.8% of participants). In other words, participants who held a goal to solve the problem during the distraction period were more accurate than participants who were merely distracted without such a goal.

#### *N*-Back Performance

To ensure that an equal amount of conscious attention was devoted to the distraction task in both the UT and MD conditions, we analyzed performance on the *n*-back task between the two groups. In the *n*-back, participants had to press a space bar whenever a digit appeared on the screen that appeared 2 trials ago (e.g., 3, 7, 3). During this task there were 14 trials that required a response (hit) and 76 trials that did not (responses here are “false alarms”). The number of hits did not differ between the UT (*M* = 11.91, *SD* = 2.49) and MD (*M* = 11.13, *SD* = 3.28) conditions, *t*(134) = 1.557, *p* = 0.122. Likewise, false alarm rates for the UT (*M* = 4.91, *SD* = 11.78) and MD (*M* = 3.32, *SD* = 4.04) conditions did not differ, *t*(82.56) = 1.05, *p* = 0.296.^6^ Thus, participants’ performance on the distraction task was comparable across the two conditions, and therefore cannot account for the performance differences on the logical reasoning problem.

### Discussion

Previous research has largely studied unconscious thought in the judgment of affectively laden (positive/negative) information such as the preference for cars, roommate, or apartments (Dijksterhuis, 2004; Dijksterhuis et al., 2006; Bos et al., 2008; Lerouge, 2009; Ham and van den Bos, 2011; Messner et al., 2011). In contrast, the current experiment used materials from a logic problem. We presented participants with a deductive reasoning problem one conditional statement at a time, and then distracted participants from thinking about the problem while they either did (UT condition) or did not (MD condition) have a goal to eventually solve the problem. Results indicated that participants who had a goal to solve the logic problem during the distraction period chose the correct answer more frequently than those who were distracted without a goal. This finding cannot be explained by the workings of an emotion-based experiential system, because the logical reasoning problem required the use of rules which is better accomplished by the analytical rational system (e.g., Epstein, 1994; Sloman, 1996; Kahneman, 2003). Rather, the current findings uncover the possibility that unconscious thought can operate via the rational system.

The current results are, of course, preliminary. Although our sample size was substantial (68 per condition), our outcome measure was simply a one-shot answer to a multiple choice question, which certainly leaves room for error. The most conservative conclusion we can draw from these results is that unconscious thought does not appear to be redundant with processes in the experiential system, and initial evidence supports the idea that unconscious thought can operate in an analytical fashion.

## General Discussion

Complex judgments and decisions require careful consideration and thought. Extensive debate has surrounded the specific type of thought that is best suited for such a task, and analytical/rational and emotional/experiential modes of processing have played central roles (e.g., Epstein, 1994; Sloman, 1996; Kahneman, 2003; Evans, 2008; Mikels et al., 2011; Usher et al., 2011). Unconscious thought is another mode of processing that has been implicated in complex judgments and decisions (Dijksterhuis, 2004; Dijksterhuis and Nordgren, 2006; Dijksterhuis et al., 2006; Dijksterhuis and Strick, 2016), yet has been confounded both theoretically (e.g., dual-process theories) and empirically with fast, experiential, intuitive, and affective processes. The current research tested the hypothesis that unconscious thought is independent from processes in the experiential mode as proposed by CEST, and explored the possibility that unconscious thought can be rational. Results support the existence of a UTE and UTA in complex information processing, and also provide evidence that unconscious thought is distinct from the experiential and rational modes, and can be analytical.

### Evidence for an Unconscious Thought Effect

The results of two experiments demonstrated the existence of an effect and advantage of unconscious thought over alternative forms of thinking. In Experiment 1, participants who were distracted with anagrams made superior judgments about roommates relative to participants who thought consciously about the options. This effect did not carry over to the final roommate choice, however. Experiment 2 revealed a difference between goal-directed unconscious thought and mere distraction in solving a logical reasoning problem. Thus, two separate experiments with diverse methods revealed a benefit of unconscious thinking over deliberation (Experiment 1) and mere distraction (Experiment 2) in complex information-processing.

The existence of a sophisticated unconscious mode of thought has come under scrutiny and criticism (e.g., González-Vallejo et al., 2008; Lassiter et al., 2009; Newell and Shanks, 2014; Nieuwenstein et al., 2015), but past research and a meta-analysis have found evidence for an effect (see Strick et al., 2011). The current results add to the literature by providing two well-powered experiments that reveal a positive effect of unconscious thinking (vs. conscious thought and mere distraction) on outcomes related to judgments of roommates and analytical problem solving.

We borrowed methods from previous research for Experiment 1 (Usher et al., 2011) and developed methods for Experiment 2 based on an existing UTT paradigm (e.g., Dijksterhuis, 2004). Therefore, we are confident that we manipulated mode of thought as intended and believe that the observed effects are best interpreted in light of established research paradigms and theory. In that case, the current results support the existence and advantage of a goal-directed unconscious thought process in complex judgments.

### Unconscious thought Is Not Merely Experiential

More novel was the evidence from the current research that unconscious thought is unique from experiential/emotional processing modes, and can be analytical. In Experiment 1, roommate descriptions were better integrated and processed by unconscious (vs. conscious) thought, but especially when paired with a prior rational mindset. This finding is inconsistent with the notion that unconscious thought effects are merely the result of a dominant experiential system.

Experiment 2 extended the idea that unconscious thought can operate via the rational system by testing the effect of unconscious thought in a logical deductive reasoning problem, and results revealed that unconscious thought increased the frequency of solving the reasoning problem relative to mere distraction. We take this result with caution because it is based on a single outcome and rates for solving the problem correctly were low (18.4%) among the UT and MD conditions. Yet, differences between these two conditions were significant, and in the hypothesized direction, suggesting that unconscious thought (vs. mere distraction) contributed to logical reasoning.

Components of the experiential system include affective processes and intuition, both of which have been implicated in value integration and optimal judgments of complex information (e.g., Wilson and Schooler, 1991; Bechara and Damasio, 2005; Slovic et al., 2007). Yet, the effects of unconscious thought appear to be distinct from these. Effects of unconscious thought in the current research emerged when affective processes were dampened down by a rational mindset (Experiment 1) and when the information to-be-processed was purely logical in nature (Experiment 2). Furthermore, although intuitions may arise from inaccessible and unconscious processes, they are often experienced at a conscious level as a “nudge” or “gut-level” preference for something (e.g., Price and Norman, 2008). Based on UTT, we have no reason to believe that products of unconscious thought are felt consciously. However, we did not assess subjective feelings at the time participants made their judgments, and it is therefore possible that they experienced some sort of “nudge” toward one option over the others. Nonetheless, the current research suggests that unconscious thought is not redundant with processes in the experiential system because the effects of unconscious thought emerged in non-affective domains and (presumably) without the inklings of intuition.

### Implications and Future Research

We see at least three important implications of our research findings. First, our results support the idea that equating experiential/emotional systems to fast unconscious processes, and analytical/rational systems to slower conscious processes, is a misleading overgeneralization. The evidence from the two current experiments suggests that—at least under some contexts—slow yet unconscious processes can consider information and form judgments and decisions independent of experiential or rational systems. Thus, the speed of, or the evident logical or emotional basis of, a decision or choice might not serve as a useful clue that conscious or unconscious processes produced the response. Future research and theorizing may benefit from considering alternative, or less constraining, categories of information processing and problem solving.

Second, our results challenge the idea that unconscious thought is not rule-based. Dijksterhuis and Nordgren (2006, p. 101) offer the *rule principle* in UTT which “states that conscious thought can follow strict rules and is precise, whereas unconscious thought gives rough estimates.” To be clear, research does indicate that conscious thought outperforms unconscious thought in gambling and betting tasks which require probabilistic rule-based solutions (Payne et al., 2008). Apparently, unconscious thought cannot do math. Yet, our second experiment demonstrates that unconscious thought can follow rules provided in an analytic problem, and use those rules to successfully solve the problem more frequently than the negligible thought involved in control-group participants. It is also noteworthy that the problem used in Experiment 2 involved ordering a person’s tasks in a day, a scenario with which undergraduate participants are likely more familiar than gambling tasks. All of this is to say that there are apparently some contexts in which unconscious thought can follow, or use, rules to increase the likelihood of correctly solving a problem. It will be important for theorists to carefully consider what *type* of rules unconscious thought can consider, and in what *contexts*, and for future research to investigate these possibilities.

Finally, we believe that our research, added to the growing body of research investigating the UTA and UTE, strongly implies psychological moderators to the effects of unconscious thought. Both the UTA and UTE are fairly small effects according to Strick et al.’s (2011) meta-analysis. Further, a recent meta-analysis *only* of experiments following the “multi-attribute paradigm” (e.g., Experiment 1 in which several options are described by multiple attributes), and a highly powered experiment using car options, finds no UTA using that paradigm (Nieuwenstein et al., 2015). Yet, our first experiment, powered with data from 319 participants, found a significant UTA using the multi-attribute paradigm (as have other recent, and well-powered, experiments, e.g., Manigault et al., 2015). Thus, we offer that the issue might be less about *whether* unconscious thought out-performs conscious thought, but in what contexts, or with what information.

### Limitations

The main purpose of the current research was to test the possibility that unconscious thought can benefit decision-making in analytical contexts, and ultimately test whether unconscious thought is distinct from the emotion-based experiential system. Although we present two experiments that support this possibility, these experiments are limited in their ultimate conclusions.

One limitation in Experiment 1 is that we assumed that Roommate B was the best option and Roommate A was the worst option. Although we used stimuli from past research that were constructed to ensure that one roommate had the most “objectively” desirable traits (and one had the least), and were pre-tested on levels of importance to exclude extreme attributes (see Usher et al., 2011), we do not know the *subjective* importance participants assigned to each trait. For instance, personality may be more or less important than cleanliness or sense of humor to a certain individual. Nonetheless, these attributes were used in prior research (Usher et al., 2011, Experiments 3 and 4) and based on numerous other UTT experiments with multi-attribute decision paradigms (see Dijksterhuis, 2004; Bos et al., 2008; Dijksterhuis et al., 2006; Ham et al., 2009; Usher et al., 2011; Manigault et al., 2015).

On a related note, the first cycle of roommate attribute presentation could be conceptualized as a “first impression,” and we did not fully balance the valence of these initial presentations. Specifically, the best option “B” initially displayed three negative and one positive attributes, the worst option “A” displayed two positive and two negative attributes, and the middle option “C” displayed two positive and two negative attributes (see Appendix A). Although the relative number of positive and negative attributes varied with each cycle, the first cycle may have had a relatively stronger influence on impressions. Even though the best option “B” was negatively skewed in this initial presentation, it was still rated most favorably overall. So, if a bias existed early on it did not appear to influence overall roommate judgments.

Additionally, we did not have a manipulation check for the mindset manipulation in Experiment 1. Although this manipulation has been used successfully in past research (Krauss et al., 2004; Usher et al., 2011), and all of the current participants complied with instructions, a measure of relative reliance on the rational vs. experiential mode would have made our conclusions stronger. Likewise, a measure of dispositional reliance on the experiential vs. rational system (i.e., Rational Experiential Inventory; Pacini and Epstein, 1999) would have allowed us to assess potential individual differences on this dimension.

Another limitation of the current experiments is that interpretations rely on behavioral outcomes far removed from the covert psychological processes involved in unconscious thought. Future research should take advantage of neuroimaging techniques (see Creswell et al., 2013, for example) or other techniques (e.g., thought probes) that can assess the neural or psychological mechanisms occurring during the distraction period, rather than relying solely on behavioral outcome measures.

## Conclusion

Past research has shown that judgments and decisions of complex information benefit from a period of distraction relative to deliberation or no time period. This benefit is presumed to result from goal-directed unconscious thought occurring during the distraction period (Dijksterhuis and Nordgren, 2006). Yet, it has been suggested the observed benefit from distraction is not due to unconscious thinking *per se* but instead to the emotion-based experiential system, which is presumably strengthened during a period of distraction (relative to deliberation). We replicated past research and systematically manipulated a reliance on the experiential vs. rational system (Experiment 1) and discovered that a period of distraction facilitated outcomes independently from these two processing modes (and to a greater degree when paired with the rational mode). Thus, a dominant experiential mode cannot solely account for the decision benefits observed following distraction. We attribute this benefit to an active unconscious thought, consistent with UTT. In a follow-up experiment (Experiment 2) we manipulated unconscious thought (vs. mere distraction) toward solving a logical reasoning problem and found that unconscious thought was superior at this analytical task. Taken together, these results suggest that unconscious thought can facilitate the processing of complex information, as proposed by UTT, and is not redundant with processes in the experiential system. In fact, unconscious thought may be rational.

## Ethics Statement

This project was approved by the Institutional Review Board of Montana State University. Participants were healthy, college-aged students at a public university. All participants were provided detailed information about the experiments, and consented to participation before the experiment began. Participants were allowed to withdraw at any point in time (none did). Participants were compensated with course credit for participation, and there were no risks beyond the minimal risks one experiences in everyday life. Participants were debriefed fully at the end of the experiment, allowed time to ask questions, and were given contact information for future inquiries.

## Author Contributions

IH was the graduate advisor of KG during the time of this work. As a mentoring team, KG and IH developed the idea and design of this work, collected and analyzed all data, and drafted and revised the manuscript.

## Conflict of Interest Statement

The authors declare that the research was conducted in the absence of any commercial or financial relationships that could be construed as a potential conflict of interest.

## Footnotes

1 Research using this paradigm typically ensures that the “best” option is more desirable than the “worst” option. For example, attributes that are extremely positive or negative are excluded, or attributes across options are positive/negative versions of the same trait (e.g., “Car A gets good gas mileage” “Car B gets poor gas mileage”; see Dijksterhuis, 2004; Dijksterhuis et al., 2006; Bos et al., 2008; Ham et al., 2009; Usher et al., 2011; Manigault et al., 2015). The attribute stimuli used in the first experiment followed these parameters.

2 We used the analytical vs. intuitive mindset manipulation from Usher et al.’s (2011) Experiment 2.

3 Due to a programming error, we failed to collect demographic information.

4 Anagrams were pre-tested for an optimal word-length to ensure they were solvable in about 20 s. Participants had the option to skip ahead if they could not find the solution.

5 http://www.lsac.org/jd/lsat/prep/analytical-reasoning

6 Degrees of freedom are corrected due to a violation in the assumption of equality of variances.
