# Supplementary material for: Not Merely Experiential: Unconscious Thought Can Be Rational
Source: Front Psychol. 2017 Jul 6;8:1096. doi: 10.3389/fpsyg.2017.01096 (PMC5498519; doi:10.3389/fpsyg.2017.01096)
Supplement: Supplementary file 1 [file Data_Sheet_1.pdf]

Appendix A

Roommate A	Roommate B	Roommate C
Has good income	Has different tastes than you	Has a sense of humor
Has different tastes than you	Has nice friends	Has a variety of interests
Has a variety of interests	Sometimes leaves dirty dishes in the sink	Has poor income
Is a bit uptight	Lacks a sense of humor	Is dull to be with
Is a bad cook	Is a relaxed and easygoing person	Plays unpleasant music while at home
Has nice friends	Plays pleasant music while at home	Has unkempt physical appearance
Sometimes leaves dirty dishes in the sink	Takes care of his/her physical appearance	Has low grades in school
Has good grades in school	Is fun to be with	Has boring friends
Is dull to be with	Gets good grades in school	Is a bad cook
Has a sense of humor	Lacks a variety of interests	Keeps the kitchen clean
Plays pleasant music while at home	Has a good income	Has similar tastes to you
Has an unkempt physical appearance	Is a good cook	Is a bit uptight
